# Draft Genome Sequences of *Clostridium* Strains Native to Colombia with the Potential To Produce Solvents

**DOI:** 10.1128/genomeA.00486-15

**Published:** 2015-05-21

**Authors:** Juan Pablo Rosas-Morales, Ximena Perez-Mancilla, Liliana López-Kleine, Dolly Montoya Castaño, Diego Mauricio Riaño-Pachón

**Affiliations:** a Institute of Biotechnology (IBUN), Universidad Nacional de Colombia, Bogotá D.C., Colombia; b Statistics Department, Sciences School, Universidad Nacional de Colombia, Bogotá D.C., Colombia; c Brazilian Bioethanol Science and Technology Laboratory (CTBE), Brazilian Center for Research in Energy and Materials, Campinas, Brazil

## Abstract

Genomes from four *Clostridium* sp. strains considered to be mesophilic anaerobic bacteria, isolated from crop soil in Colombia, with a strong potential to produce alcohols like 1,3-propanediol, were analyzed. We present the draft genome of these strains, which will be useful for developing genetic engineering strategies.

## GENOME ANNOUNCEMENT

The need for obtaining alternative energy sources has renewed the interest in the production of different biological chemicals and biofuels using inexpensive substrates and microorganisms like *Clostridium* (1). Researchers have thus joined forces for evaluating the use of several by-products in alternative technologies, like the use of lignocellulosic material to produce acids and alcohols, or microbial fermentation of glycerol to produce 1,3-propanediol (1,3-PD) (2, 3). The latter is extremely relevant as it is the monomer from a polymer of commercial interest known as polytrimethylene terephthalate (PTT) (4).

We have previously shown that several strains from our collection have promising total solvent yield from glucose and the ability to hydrolyze different polysaccharides (5). Some of them have also shown great potential to produce higher yields of 1,3-PD from glycerol than reference strains (6). The genomes of four of these strains isolated from crop soils (7), named *Clostridium* sp. IBUN 13A (potatoes), 62F (grass), 125C (soya), and 22A (potatoes), were sequenced in an Illumina HiSeq2000 instrument using the NExtera library preparation kit producing 3,211,515 paired-end sequences (2 × 101 bp) per strain on average. Preprocessing of reads was done with Clean_reads and Fastx-toolkit v0.0.14. Genome assembly was performed with Velvet v1.2.10 (8), and postassembly improvement was achieved using the tools IMAGE (9) and iCORN (10) from PAGIT toolkit (11).

Prediction of RNA genes was carried out with tRNAscan v1.21 for tRNA (12) and RNAmmer v1.2 for rRNA (13). Protein-coding genes were predicted using the MAKER2 pipeline (14), employing Glimmer v3.02 (15), GenemarkS v4.17, and Prodigal v2.6 (16) as *ab initio* gene predictors. The function assignation for the predicted genes was done using the RAST annotation server. Information about the genome of these four strains is summarized in Table 1.

**TABLE 1:** Summary of the *Clostridium* sp. draft genomes

Strain	Assembly length (bp)	% G+C content	No. of contigs	No. of genes	No. of proteins	Accession no.
IBUN13A	4,643,590	28.65	261	4,086	4,022	JZWG00000000
IBUN62F	3,836,807	28.77	85	3,356	3,288	JZWH00000000
IBUN125C	4,596,888	28.67	71	3,959	3,888	JZWF00000000
IBUN22A	4,607,385	28.67	208	4,124	4,063	JZWE00000000

All genes implicated in the reductive pathway for 1,3-PD production, including the two-component regulatory system, were found grouped in the same contig for three strains. In strain 62F, those protein-coding sequences were not detected in agreement with phenotypic observations shown in other studies (6). Hydrolases implied in polysaccharide degradation were identified in all strains, highlighting their biotechnological potential.

Metabolic reconstruction and gene finding from these genomes will lead to the development of a genetic engineering strategy for the increase of 1,3-PD production.

### Nucleotide sequence accession numbers. 

The draft genome sequences of the four strains have been deposited at DDBJ/EMBL/GenBank under the accession numbers given in Table 1. In this paper, the first versions of the genomes are described.
